# Congenital orbital teratoma: a case report with preservation of the globe and 18 years of follow-up

**DOI:** 10.1186/s12886-021-02229-2

**Published:** 2021-12-28

**Authors:** Panagiotis A. Tsoutsanis, George C. Charonis

**Affiliations:** 1 Ipswich Hospital, Ipswich, UK; 2 Athens Vision Eye Institute, Athens, Greece

**Keywords:** Orbital teratoma, 18-year follow-up, Proptosis, Fetal surgery

## Abstract

**Background:**

Congenital orbital teratomas are extremely rare, usually benign neoplasms, comprised of cells originating from all three germ cell layers. Clinically the tumor appears solid, most of the times is intraconal and presents as a rapidly growing mass leading to a massive unilateral axial proptosis, chemosis, exposure keratopathy, markedly distended eyelids and often, loss of vision. To prevent these complications, tumor excision usually involves enucleation or even orbital exenteration.

**Case presentation:**

We report a case of a 1-day old infant who presented with dramatic proptosis at birth due to a true congenital orbital teratoma. We describe the clinical findings, the preoperative neuroimaging, the surgical management which included complete tumor resection with preservation of the globe to allow for optimal orbital growth, the histopathological evaluation, and the clinical course during 18 years of follow up.

**Conclusion:**

Every effort to salvage the globe should be made to achieve the best possible orbito-facial development. Furthermore, the value of prompt surgical management with a less invasive transconjunctival globe sparing procedure can be appreciated in our case.

## Background

A teratoma is defined as a congenital neoplasm that contains elements deriving from all three germinal cell layers (ectoderm, mesoderm, endoderm). Teratomas represent about 6–10% of pediatric tumors, most commonly located in the testes, ovaries or the retroperitoneum [1]. Congenital orbital teratomas are exceedingly rare but have the potential to cause massive proptosis that needs urgent management.

## Case presentation

**Fig. 1:**
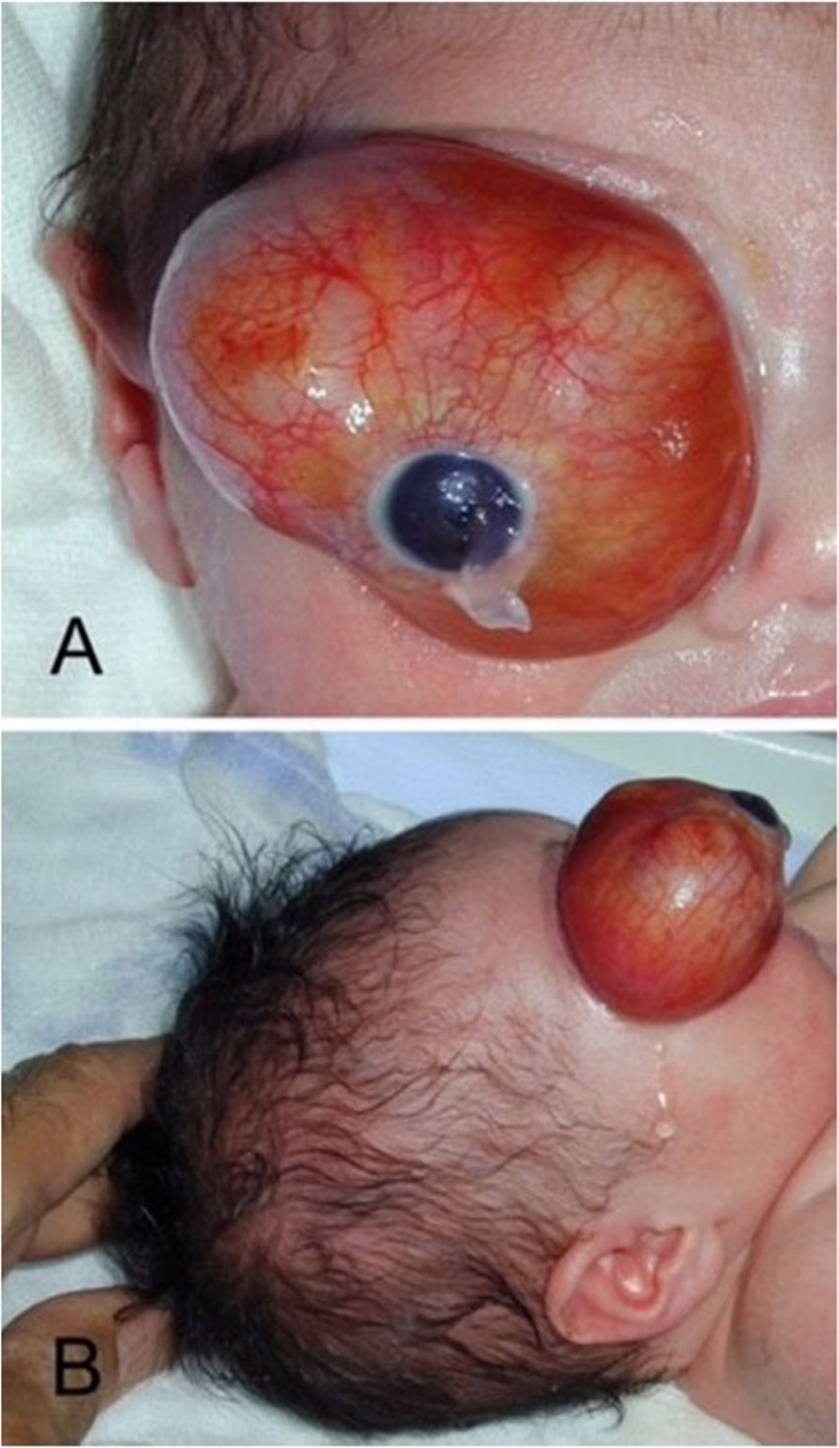
**a** and **b** The 1-day infant at presentation

A 1-day old female newborn presented with severe unilateral proptosis (Fig. 1 a, b). She was born at term with elective caesarian section after an uncomplicated pregnancy. The 25-year-old Caucasian mother had a normal antenatal history, no history of recreational drug use, no pharmacologic therapy or known exposure to chemicals or radiation.

Ophthalmologic examination revealed massive proptosis of the right eye and an absent direct or consensual pupil reaction of the right eye. Despite the degree of proptosis, possibly due to the very early examination of the newborn immediately postpartum, the cornea was clear. The anterior segment was examined via a portable slit lamp and showed neovascularization of the iris without hyphema. The lens was clear and there was a good red reflex of the posterior segment.

**Fig. 2:**
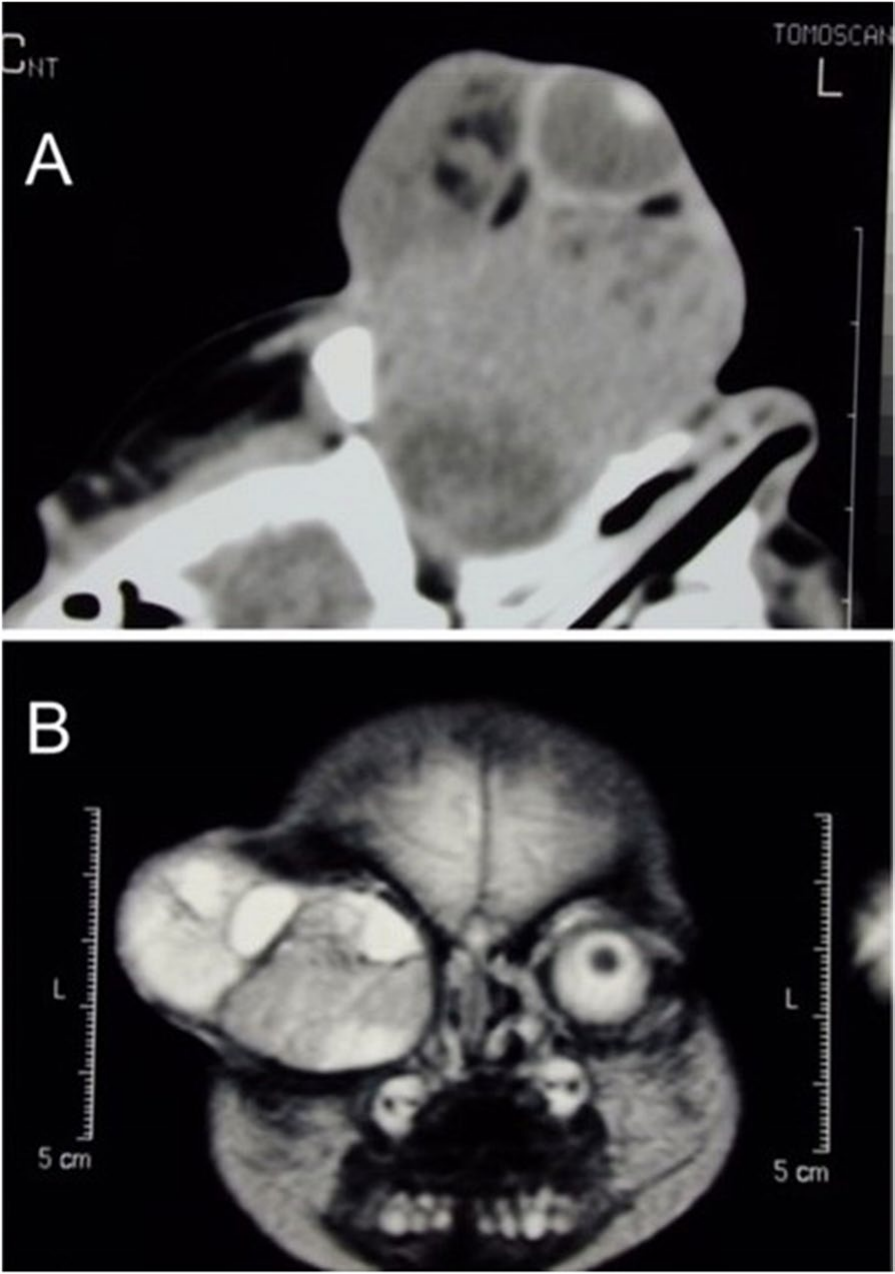
**a** CT of the orbits shows a large heterogeneous mass, filling the entire orbit. **b** T2-weighted MRI shows the tumor to be hyperintense to fat and extraocular muscles

A CT of the brain and orbits showed a large heterogeneous mass arising entirely in the right orbit, engulfing the globe and the optic nerve, and filling the entire orbit. No bony erosion or calcifications were noted (Fig. 2a). T2-weighted MRI imaging shows predominantly the tumor to be hyperintense to fat and extraocular muscles (Fig. 2b).

**Fig. 3:**
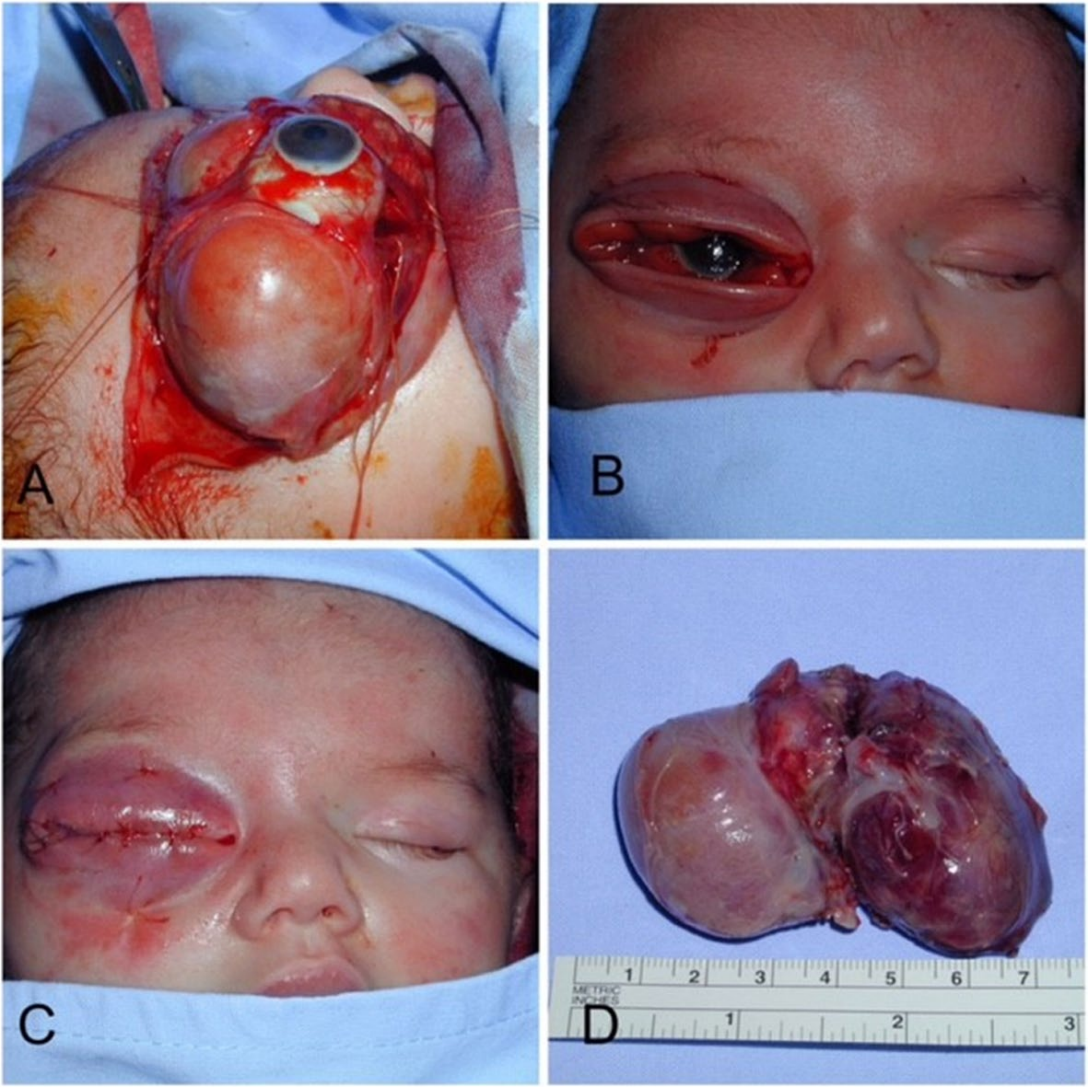
**a** Intraoperative photo showing the lesion just prior to lateral rectus disinsertion. A dissectible plane can be seen. **b** After tumor removal, significant eyelid laxity is noted. **c** Upper and lower fornix sutures were placed and the laxed tissues were allowed to retract to their normal size over time. **d** The excised tumor is seen

Due to the degree of proptosis and the imminent risk for severe exposure keratopathy and corneal breakdown, urgent surgical excision of the tumor was scheduled. One day later, the tumor was removed completely via a 360-degree conjunctival limbal peritomy, identification of the 4 recti muscles and temporary disinsertion of the lateral rectus muscle in order to provide the necessary dissection plane to allow for complete tumor excision (Fig. 3a).

After tumor removal there was significant upper and lower eyelid laxity and conjunctival prolapse due to an expansion effect on the eyelid and periocular tissues exerted by the tumor (Fig. 3b). However, it was decided not to sacrifice eyelid or conjunctiva tissue at this time and a decision to place upper and lower forniceal sutures and a reversible suture tarsorrhaphy and allow the tissues to retract to their normal size was made, (Fig. 3c). Since the prognosis for vision in that eye was very poor, it was decided to keep the tarsorrhaphy sutures for 3 weeks in order to help with the healing process and keep the “redundant” and laxed tissues in place. The eyelid tone and the conjunctival fornices were restored during this time. The tumor measured 7X5X4.5 cm (Fig. 3d).

**Fig. 4:**
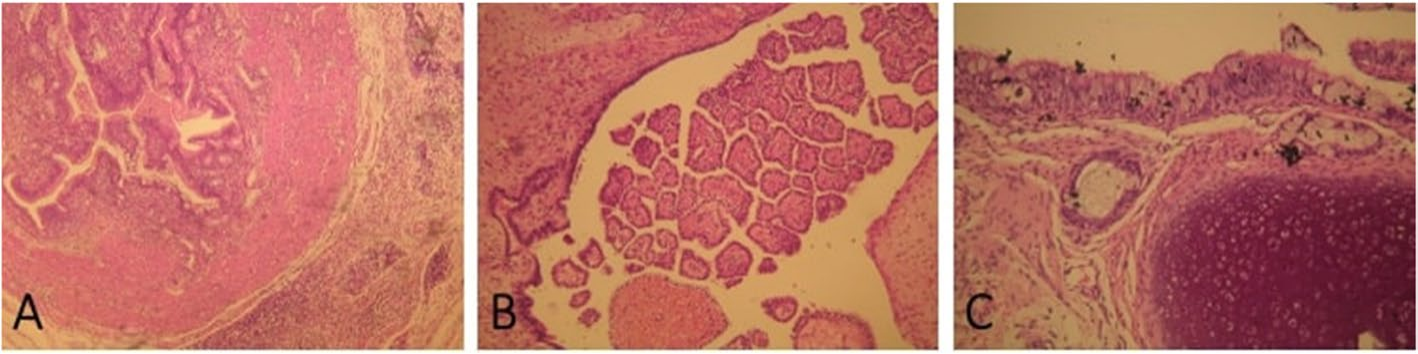
**a** H + E stain of the tumor showing a mature teratoma containing cells from three of the intestinal wall’s layers, the smooth muscle layer, the submucosa and the columnar epithelium of the mucosa**. b** Cells forming a choroidal plexus papilloma is seen**. c** Respiratory epithelial cells alongside bronchial glands and cartilage tissue is depicted

The specimen was stained using a hematoxylin and eosin dye. Figure 4a shows the mature teratoma containing cells coming from three of the intestinal wall’s cell layers, namely the smooth muscle layer, the submucosa, and the mucosa with its columnar epithelium, while in Fig. 4b another portion of the specimen shows cells forming a choroid plexus papilloma. Figure 4c depicts respiratory epithelial cells alongside bronchial mucosal glands as well as bronchial cartilage tissue.

**Fig. 5:**
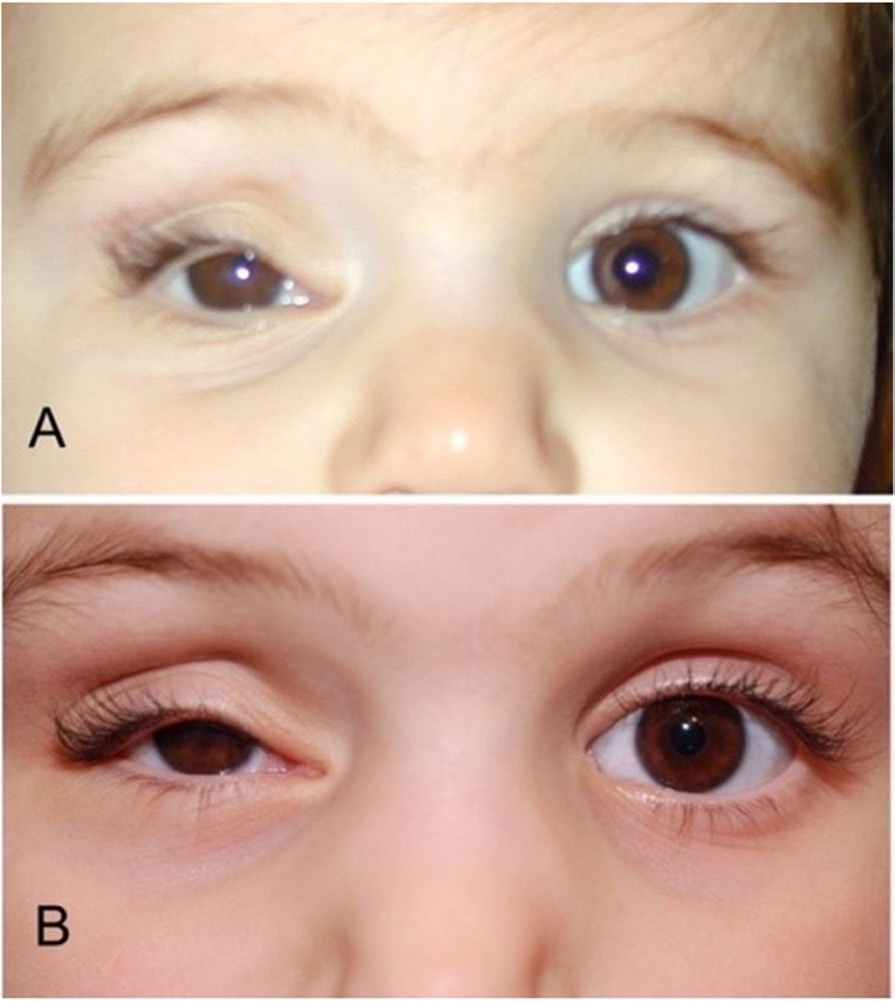
**a** The child at age 3**. b** child at age 7. There is more volume loss in the right orbit

**Fig. 6:**
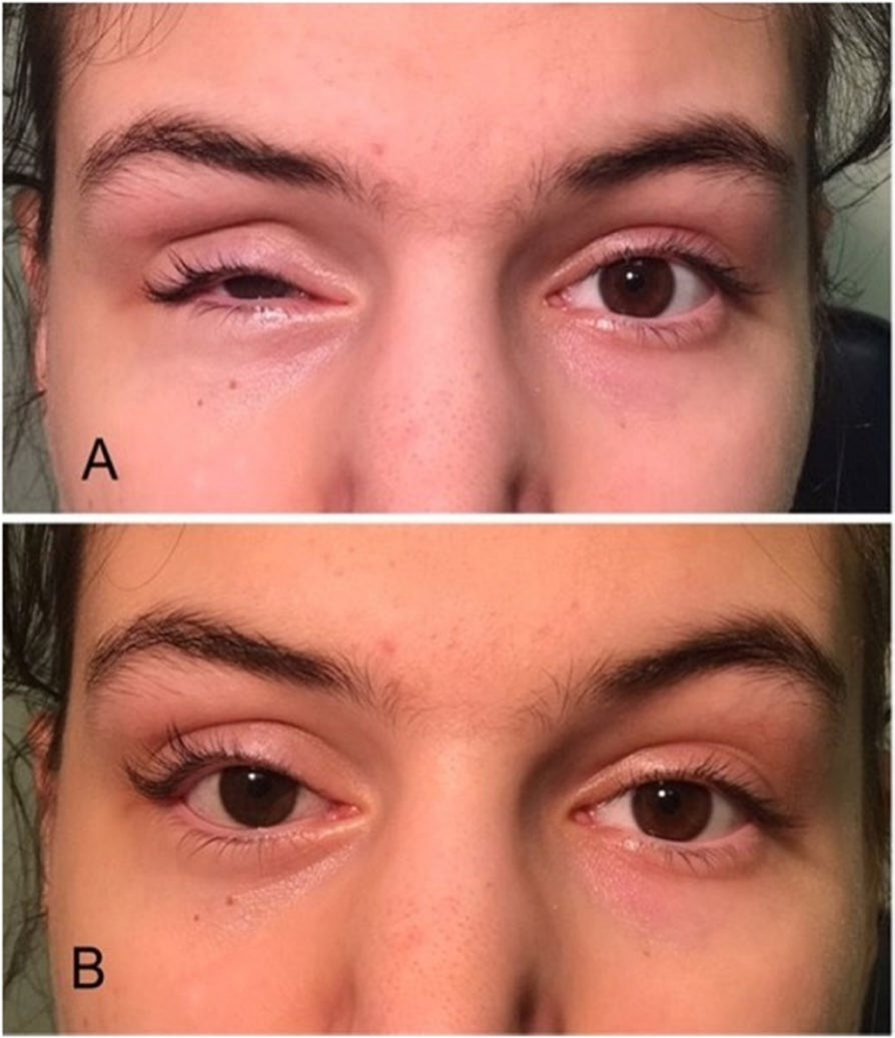
**a** Our patient at age 18. The volume loss of the orbital soft tissues has progressed but the orbital bones have developed symmetrically**. b** Patient at age 18 wearing a scleral shell prosthesis. Further aesthetic improvement to address the nasal ptosis has been suggested but deferred at present time

Postoperatively, our newborn patient recovered remarkably well. Unfortunately, as expected due to the compression effect and to the ischemic changes manifested by the neovascularization of the iris, there was no vision from that eye, but the orbit developed remarkably well over the next years. We decided to follow the child clinically over the next 18 years and to proceed with orbital imaging only if required by the clinical exam findings. Our decision was based on complete tumor removal with clear margins, on the very low malignant potential and on the requirement for general anesthesia during imaging. The anterior segment hypoxia reversed as shown from the absence of iris neovascularization. There was a progressive mild to moderate volume deficit of the right orbit manifesting mainly as a deepened superior sulcus and a corresponding upper eyelid ptosis/contour abnormality (Fig. 5a, b). When our patient was mature enough to wear a scleral shell, a custom-made shell was fitted. The family elected not to undergo any further surgery or minimally invasive procedures for cosmetic reasons at this point. Figure 6a and b shows our patient currently at age 18.

There was no recurrence or malignant transformation during this 18-year follow-up period and our patient graduated from high school and was recently admitted to Law school.

## Discussion and conclusion

### Epidemiology

The word teratoma comes from the Greek and means monster. In about 1 out of 1000 cases, teratomas can present in the orbit [2], comprising the true congenital orbital teratomas which represent between 0.8–1.3% of orbital tumors in childhood. The first unquestionably reported congenital orbital teratoma was by Broer and Weigert in 1879 [3]. Since then, there have been 70 published cases that fulfill the characteristics of a true congenital orbital teratoma [4]: presentation at birth, being primarily intraorbital and comprising of all 3 germ cell layers. Some authors have found a female predominance (F/M:2/1) [5] or left orbit predominance [6] but others have failed to show this discrepancy [3]. Heredity and teratogens have not been shown to play a role in the incidence as there is no family history of congenital deformities with nonconsanguineous parents, there is a history of normal pregnancy and delivery and no history of teratogenic influences to the mother [7].

Orbital teratomas are usually benign and several theories regarding the origin of their pathogenesis exist. One of the most widely accepted theories pinpoints abnormal migration of pluripotent cells as the cause of such tumors, as such migration introduces these cells to the orbital area where they escape their inhibitory regulation and thus proliferate in an unregulated fashion [8, 9].

The malignancy rate for all teratomas has been estimated at around 21% [10]. Malignancy is considered when the tissue is embryonal or immature. The prognosis for immature teratomas is related to the age, site, grade of immaturity and is generally good if complete excision is performed early in life. The vast majority of orbital teratomas are benign [11].

### Presentation

The most common presentation of orbital teratomas is that of a well circumscribed rapidly growing mass causing massive unilateral axial proptosis, chemosis, exposure keratopathy and markedly distended eyelids with elongation of the palpebral fissure. In primary congenital orbital teratomas, the orbital enlargement is not accompanied by bony destruction, lysis or intracranial or sinus involvement and the optic nerve is usually either encased within the tumor, adherent or severely compressed leading to severe ischemia and atrophy [2, 6, 11].

Clinically the tumor appears solid, has no bruits and shows variable degrees of transillumination. Most tumors are intraconal, are confined by the 4 recti muscles and, thus, appear quadroangular in shape. The eye is usually buried within the large tumor and shows normal architecture but the motility is markedly limited [3].

### Investigations & Diagnosis

Sonography shows heterogeneous signals of high and low internal reflectivity and multiple cystic spaces. CT scan often demonstrates a well-circumscribed mass with focal calcifications and no osseous destruction or lysis. MRI shows a heterogeneous mass with varying signal intensities having hyperintense and hypointense areas with respect to the opposite orbital fat [5]. Furthermore, MRI is indicated to exlude intracranial extension and help during the preoperative evaluation for complete tumor excision [3].

Definite diagnosis of orbital teratoma can only be made with biopsy followed by histopathological analysis, showing, ideally, cells of all three germ cell layers. However, the apparent lack of one germ cell layer may result from sampling errors and should be considered in the pathologic diagnosis [3]. The most dominant cells in orbital teratomas are those of ectodermal origin, followed by those of mesodermal origin and at last those of endodermal [5, 11].

Differential diagnoses of orbital teratomas include rapidly growing lesions of the neonatal period: dermoid cyst, epidermoid inclusion cyst, hemangioma, lymphoma, microphthalmos with cysts, congenital cystic eyeball, orbital hematoma and malignant tumors (neuroblastoma, rhabdomyosarcoma, retinoblastoma and other metastatic tumors) [2, 6, 12].

Although not many cases have been reported in literature, antenatal screening of congenital orbital teratomas is possible. The screening tool of choice is that of ultrasound which can detect a mixed sonographic image made up of cystic and solid complex masses with significant vascularization. If further study regarding the location, extent and nature of the tumor is needed, a fetal MRI can be considered [6].

### Treatment options

Fortunately, most of the congenital orbital teratomas are benign and therefore there is no need for post operative adjuvant radiotherapy and/or chemotherapy. Its prognosis is considered very good if treated early in the postnatal life, especially if complete surgical excision is achieved. Due to the fact that there have been a few cases of recurrence and, anectodically, of malignant transformation, there is the need of alertness and regular follow ups, ensuring that should a recurrance occurs, it will be addressed promptly [6].

Treatment is by complete surgical excision, if possible. Due to the massive proptosis on presentation, the surgery is usually carried very early postpartum. The goal of early treatment is primarily to try and save the vision of the affected eye, if possible and, secondarily, to salvage the globe. Unfortunately, due to the massive proptosis and the propensity of the tumor to encase or adhere to the optic nerve, there is irreversible ischemic damage even at the earliest possible postpartum intervention. Our case had a nonreactive pupil and evidence of severe anterior segment ischemia manifesting as iris neovascularization when the child was examined at 24 h postpartum.

Every effort should be made to salvage the globe. Even when the outlook seems dismal for globe preservation due to the dramatic clinical presentation, our case demonstrates that complete en bloc excision of the tumor was possible with a transconjunctival 360- degrees limbal peritomy approach without the need to rely on more aggressive orbital or craniofacial procedures. Disinserting one of the recti muscles provides enough space to dissect safely around the tissue plane of the tumor. Another learning aspect from this case is to avoid the urge to sacrifice the stretched, “redundant” upper and lower eyelid and conjunctival tissue that is often present after tumor removal. Placing full thickness forniceal sutures to tuck the conjunctiva and a reversible suture tarsorrhaphy and allowing the eyelid tissues to retract into their normal dimension and tone avoids further unnecessary iatrogenic trauma and may yield a better postoperative outcome.

Review of the most recent literature (post-1990) showed a tendency towards more aggressive surgery (orbital exenteration) than globe sparing surgery [3, 9, 13–18].. The reasons for choosing exenteration over globe preservation, ranged from lack of resources to absence of an organized eye or an optic nerve due to rapid tumor growth [6–8, 11, 12, 19–25].

### Outcome & follow-up

Globe sparing surgery encourages adequate orbito-facial development leading to better facial symmetry and improved cosmesis. The fitting of a custom-made scleral shell is easier than the handling and the aesthetics of the larger, heavier, and less physiological orbito-facial prostheses that are typically used after orbital exenteration especially in such young patients. Another disadvantage of orbital exenteration surgery is that, quite often, further reconstructive craniofacial surgery and rehabilitation depending on midfacial growth is required [26].

An interesting point about our case is the significantly long postoperative follow up period that we provide. The patient’s facial development can be followed at different ages, providing valuable feedback on how the periorbital tissues develop over the years after tumor excision. There has been no recurrence, the postoperative period has been uneventful, and the overall aesthetic result is reasonable. The relative orbital volume loss and the contour asymmetry of the upper eyelid that was observed can be addressed with hyaluronic acid gels and/or upper eyelid surgery for further improvement in cosmesis in the future.

The value of prompt surgical management with a less invasive transconjunctival globe sparing procedure can be appreciated in our case.

### Learning points

- Congenital orbital teratomas are rapidly growing masses leading to massive unilateral axial proptosis, chemosis, exposure keratopathy, markedly distended eyelids and often, loss of vision.
- If treated early in the post-natal life, prognosis is considered good.
- Every effort to salvage the globe should be made to achieve the best possible orbito-facial development.
- Transconjunctival approaches with disinsertion of an extraocular muscle provide sufficient exposure to remove even massive tumors
- It is best to avoid removing “redundant” eyelid and conjunctival tissue after tumor removal and allow the tissues to retract into their normal dimensions over time.

## Data Availability

The data and images used or analysed during the current study available from the corresponding author on reasonable request.

## Abbreviations

CT scan: Computerized tomography scan
MRI scan: Magnetic resonance imaging scan
